# The degree of food processing is associated with anthropometric measures of obesity in Canadian families with preschool-aged children

**DOI:** 10.3389/fnut.2022.1005227

**Published:** 2022-09-23

**Authors:** Rahbika Ashraf, Alison M. Duncan, Gerarda Darlington, Andrea C. Buchholz, Jess Haines, David W. L. Ma

**Affiliations:** ^1^Department of Human Health and Nutritional Sciences, University of Guelph, Guelph, ON, Canada; ^2^Department of Mathematics and Statistics, University of Guelph, Guelph, ON, Canada; ^3^Department of Family Relations and Applied Nutrition, University of Guelph, Guelph, ON, Canada

**Keywords:** ultra-processed foods, NOVA food classification system, obesity, BMI, ASA24, children, health, disease prevention

## Abstract

Adopting a healthy diet remains central for the prevention of obesity. In adults, higher intake of ultra-processed food is associated with a greater risk of overweight and obesity. However, little is known about the degree of food processing and its association with anthropometric measures in families with preschool-aged children, a critical period for the development of dietary patterns. This cross-sectional study included preschool-aged children (*n* = 267) between 1.5 and 5 years of age and their parents (*n* = 365) from 242 families enrolled in the Guelph Family Health Study. Dietary assessment was completed using ASA24-Canada-2016. Foods and beverages were classified based on their degree of food processing using the NOVA Classification (unprocessed or minimally processed foods, processed culinary ingredients, processed foods, and ultra-processed foods). Associations between the energy contribution (% kcal) of each NOVA category and anthropometric measures were examined using linear regression models with generalized estimating equations, adjusted for sociodemographic variables. The energy contribution of ultra-processed foods was the highest relative to the other NOVA categories among parents (44.3%) and children (41.3%). The energy contribution of unprocessed or minimally processed foods was 29.1% for parents and 35.3% for children, processed foods was 24.0% for parents and 21.3% for children, and processed culinary ingredients was 2.6% for parents and 2.1% for children. Ultra-processed foods (% kcal) were positively associated with BMI (β = 0.04, 95% CI: 0.01–0.07, *P* = 0.02), waist circumference (β = 0.11, 95% CI: 0.03–0.18, *P* = 0.008) and body weight (β = 0.13, 95% CI: 0.03–0.22, *P* = 0.01) in parents, but not children. Unprocessed foods (% kcal) were negatively associated with waist circumference in parents (β = −0.09, 95% CI: 0.18–0.01, *P* = 0.03) and children (β = −0.03, 95% CI: 0.05–0.01, *P* = 0.01), as well as body weight (β = −0.12, 95% CI: 0.23–0.00, *P* = 0.04) in parents. The degree of food processing primarily influenced anthropometric outcomes in parents. Nevertheless, diets of children were similar, suggesting that such exposure in families may eventually lead to outcomes observed in parents.

## Introduction

Obesity is a public health problem and the increased prevalence of obesity worldwide is driven in part by changes in the global food system, replacing dietary patterns based on home-prepared foods with industrially processed and pre-packaged foods (1). Evidence suggests that a greater intake of ultra-processed foods is associated with obesity and related cardiometabolic outcomes (2, 3). Ultra-processed foods are defined by the NOVA food classification system as industrial formulations of ingredients derived from additives and food substances (4). The NOVA system is a diet classification tool that considers the nature, purpose and extent of food processing when classifying foods and beverages into four categories that range from least to most processed and include: unprocessed or minimally processed foods (e.g., whole foods, fruits, vegetables, eggs, and milk); processed culinary ingredients (e.g., sugar, salt, butter, and cooking oil); processed foods (e.g., salted nuts, simple breads, and cheese); ultra-processed foods (e.g., pre-packaged meals and breads, sugary drinks and sweetened or salty snacks) (4). Ultra-processed foods are typically energy-dense and characterized as having poor nutritional content, including higher levels of sodium, free and added sugars and saturated fats, compared to their unprocessed or minimally processed counterparts (4).

Data from household food purchases reveal an increase in ultra-processed food sales globally (5). This rise in household availability of ultra-processed foods parallels the increased prevalence of overweight and obesity (5, 6). Clinical and observational studies have also identified a potential association between ultra-processed food consumption and increased obesity risk. For example, results of a randomized controlled trial demonstrated that an ultra-processed diet caused weight gain in adults relative to an unprocessed diet despite being matched for calories (7). Cross-sectional studies reported similar findings linking higher intakes of ultra-processed foods to increased prevalence of obesity in both adults and children (8–12). Similar associations between ultra-processed food intakes and anthropometric measures or related risk markers have been identified in longitudinal studies in adults (13–17) and children (18–20). Recent systematic reviews and meta-analyses also confirm the overarching finding that ultra-processed foods are positively associated with excess body weight and obesity in adolescents and adults (21, 22) and additional factors related to cardiometabolic risk in children and adults (23). However, the role of the different degrees of food processing (ranging from unprocessed to ultra-processed) on indicators of obesity in children remains unknown.

Further, although children are the leading consumers of ultra-processed foods (24–26), few studies have explored the association between the degree of food processing and obesity risk in preschool-aged children. The focus on young children is important as early dietary behaviors may track into adulthood, potentially playing a role in the development of chronic diseases later in life (27). The home food environment also influences the development of early dietary patterns, underscoring the importance of research within the family unit (27–29). Thus, assessing dietary intake in families, including both parents and children may provide unique perspectives of the role of the family environment in shaping children’s food choices and dietary habits. Since the most effective programs for addressing and preventing childhood obesity are family based, insights into the dietary intakes of parents and children may provide the basis of future diet and weight-related behavior change interventions (30). Therefore, the aim of this study was to investigate the associations between food intake according to the degree of food processing and anthropometric indicators of obesity in Canadian preschool-aged children and their parents.

## Materials and methods

### Study design and participants

The Guelph Family Health Study (GFHS) is a longitudinal health promotion study investigating early life risk factors for obesity and chronic diseases in families with young children. This cross-sectional study collected data from families participating in the GFHS between April 2017 and March 2020. Families were recruited through the local Family Health Team, Community Health Centre, and Ontario Early Years Centres if they had at least one child between the ages 1.5–5 years, resided in Guelph-Wellington in Ontario, Canada with no plans of relocating within next year, and could respond to questionnaires in English. Parents provided written informed consent and the University of Guelph Research Ethics Board approved the study (REB #17-07-003).

A total of 246 families (749 participants; 427 parents, 322 children) were enrolled in the GFHS. Of these, 117 participants were excluded from the current analyses due to the following: missing dietary data (37 parents, 28 children), implausible energy intakes (>1.5 times the interquartile range below the 25th or above the 75th percentiles; nine parents, 17 children), pregnancy or breastfeeding (16 mothers), breastfed (nine children), as intake amounts could not be verified, and illness (one child). The final sample for this study included *n* = 365 parents and *n* = 267 children from 242 families.

### Dietary assessment

Parents reported dietary intake for themselves and parent 1 (defined as the first parent enrolled in the study, 90% mothers) reported dietary intake on behalf of their participating child(ren). Dietary intake data was evaluated for energy intakes using the National Cancer Institute’s Automated Self-Administered 24-h (ASA24) Dietary Assessment Tool, version 2016 adapted for use in the Canadian population. The ASA24 is a self-administered, web-based 24-h dietary recall program that has been validated for use among adults (31) and preschool-aged children (32). The ASA24 was derived from the USDA’s Automated Multiple-Pass Method (AMPM), providing a modified approach to traditional interviewer-administered 24-h recalls. The ASA24 uses food images to assist respondents with portion size estimation and provides nutrient content for foods, beverages, and supplements.

### Classification of foods by the degree of processing

Foods reported in the 24-h recall were manually classified according to the degree of processing according to the four NOVA classification system categories: unprocessed or minimally processed foods, including naturally present “fresh” or “whole” foods altered by methods that do not require the addition of substances such as salts, sugars, oils or fats (e.g., fresh, frozen or dry fruits and vegetables, packaged grains, legumes, fresh or frozen meat and fish, eggs and plain milk); processed culinary ingredients, found in home kitchens to cook/season foods and make dishes palatable (e.g., starch, table sugar, salt, lard, butter, and oils); processed foods, described as products made by adding processed culinary ingredients such as salt, sugars and/or oils to unprocessed or minimally processed foods (e.g., artisan breads and cheeses, canned fish, salted meat, fruit preserves and vegetables in brine); ultra-processed foods, defined as ready-to-eat food products made with industrial formulations of ingredients and additives, containing minimal or no whole foods (e.g., soft drinks, sweetened or salted pre-packaged snacks, sweetened breakfast cereals, mass-produced breads, processed meats and ready-to-eat frozen or shelf-stable meals) (4, 33, 34). This work was completed by one trained researcher using a pre-constructed standard operating procedure that was independently reviewed by multiple researchers.

Automated Self-Administered 24-h “Food Description” and “Food Source” variables were used to identify the degree of food processing by providing information about the preservation processes (e.g., fresh, frozen, dried or canned in own juice, oil, water or syrup), production methods (e.g., home, bakery, or industrially prepared), addition of ingredients (e.g., sweetened, salted, or unsalted) and source of foods (e.g., fast food or grocery store). In cases of ambiguity, food items were coded under the lesser processed category. Zero-calorie foods (e.g., water) were not classified and excluded from the analyses. Since the reporting of recipes was not required in ASA24, home-made mixed dishes were classified as “un-disaggregated home-made dish” under the unprocessed or processed foods categories, depending on the processing level of the core ingredients (35). Energy intake from foods was quantified and reported in either absolute (kcal/day) and/or relative values (% kcal/day).

### Anthropometric indicators of obesity

Anthropometric measures, including body weight, height and waist circumference were obtained at the University of Guelph Body Composition Laboratory. The measurements were performed by trained research staff and under standard conditions, with participants standing and either barefoot or in socks. Body weight (kg) was measured to the nearest 0.001 kg using a calibrated electronic weighing scale (BOD POD™, COSMED USA Inc., Concord, CA, USA). Height (cm) was measured to the nearest 0.1 cm using a calibrated wall-mounted stadiometer (Seca Model 222, Mount Pleasant, SC, USA) for parents and older children or a ShorrBoard pediatric measuring board (ShorrBoard^®^, Weigh and Measure LLC., Olney, MD, USA) for younger children. Waist circumference (cm) was measured to the nearest 0.1 cm at the top of the right iliac crest using a Gulick II measuring tape (Gulick II, Country Technology Inc., Gay Mills, WI, USA). Two measures were taken for height and waist circumference; if the difference between the values was greater than 0.5 cm, a third measure was taken and the mean of the nearest two values was reported as the final value. BMI [weight (kg)/height (m)^2^] was calculated for the parents. BMI z-scores, measures of relative weight adjusted for child age and sex, were calculated for children based on the WHO Child Growth Standards using the R package “zscorer” version 0.3.1 statistical software (36).

Percent fat mass was measured by trained research staff using the BOD POD™ digital scale (COSMED USA Inc., Concord, CA, USA) for parents or during bioelectrical impedance analysis (BIA) using a Quantum IV BIA Analyzer System (RJL Systems, Clinton Township, MI, USA) for children. Participants were instructed to avoid food and drink and vigorous physical activity for 2 h (parents) or 30 min (children) prior to the assessment. Two BIA measurements were taken; if the difference between the two resistance values was greater than 5%, a third measurement was taken. Percent fat mass from the BIA assessment for children was estimated using total body water calculation by Kushner et al. (37) and hydration constants by Fomon et al. (38).

BMI or BMI z-scores for 36 participants (*n* = 11 parents and *n* = 25 children), waist circumference data for 38 participants (*n* = 8 parents and *n* = 30 children), body weight for 16 participants (*n* = 11 parents and *n* = 4 children) and percent fat mass data for 205 participants (*n* = 141 parents and *n* = 64 children) were missing and excluded from the regression analyses.

### Statistical analysis

Data were analyzed using SAS University Edition version 3.6 (SAS Institute Inc., Cary, NC, USA) (39). Linear regression models with generalized estimating equations were fitted to estimate the associations between food intake according to the degree of food processing and obesity indicators (BMI or BMI z-scores, waist circumference, body weight and percent fat mass). Generalized estimating equations were used to obtain coefficient estimates (β), 95% confidence intervals (CIs), and *P*-values that account for dependence among participants within the same family (40). Anthropometric measures (BMI or BMI z-scores, waist circumference, body weight and percent fat mass) of parents and children were independently regressed onto each processed food category, expressed as percent of total energy. Analyses were conducted separately for parents and children. Models were adjusted for variables that were identified as potential confounders including age (years), sex, annual household income (<$50,000; $50,000–$99,999; $100,000–$149,999; $150,000 or more; Did not disclose), ethnicity (White; Other, including Black, Chinese, Japanese, Korean, Latin American, Mixed ethnicity, South Asian, Southeast Asian, and West Asian; or Did not disclose) and education for parent models (no postsecondary degree; postsecondary graduate; postgraduate training), or highest level of parental education for child models.

## Results

### Participant characteristics and energy intake

Participant characteristics are reported in Table 1. Among the total sample of 365 parents and 267 children, 59% were mothers (*n* = 216) and 52% were girls (*n* = 138). The mean age was 35.7 (SD = 4.7) years among parents and 3.6 (SD = 1.2) years among children. Approximately 80% of parents and 75% of children identified as White. A total of 49% of parents (*n* = 179) reported an annual household income of $100,000 or greater and about 35% (*n* = 127) obtained postgraduate training or degrees. The mean BMI or BMI z-score value for parents and children was 26.8 (SD = 6.1) and 0.5 (SD = 0.8), respectively. The mean waist circumference was 92.8 (SD = 15.1) for parents and 51.1 (SD = 3.3) for children. The mean percent fat mass values were 29.4 for both parents (SD = 9.9) and children (SD = 5.5). The mean daily energy intake was 2211.9 (SD = 859.5) kcal for parents and 1408.9 (SD = 381.2) kcal for children.

**TABLE 1:** Participant characteristics of the Guelph Family Health Study, by age group and sex.

Characteristic	Mothers *n* = 216	Fathers *n* = 149	Parents overall *n* = 365	Girls *n* = 138	Boys *n* = 129	Children overall *n* = 267
Age (years), mean ± SD	35.1 ± 4.6	36.4 ± 4.8	35.7 ± 4.7	3.5 ± 1.2	3.6 ± 1.3	3.6 ± 1.2
BMI (kg/m^2^), mean ± SD, *n*	26.8 ± 6.6, 212	27 ± 5.3, 142	26.8 ± 6.1, 354	0.5 ± 0.8, 127^1^	0.5 ± 0.8, 115^1^	0.5 ± 0.8, 242^1^
Waist Circumference (cm), mean ± SD, *n*	90.6 ± 15.6, 213	96.1 ± 13.9, 144	92.8 ± 15.1, 357	51.1 ± 3.4, 127	51.1 ± 3.2, 110	51.1 ± 3.3, 237
Body weight (kg), mean ± SD, *n*	72.7 ± 17.6, 212	86.9 ± 17.6, 142	78.4 ± 18.9, 354	15.4 ± 2.9, 136	16.1 ± 3.2, 127	15.8 ± 3.0, 263
Fat mass (%), mean ± SD, *n*	32.5 ± 9.2, 142	24.2 ± 8.9, 82	29.4 ± 9.9, 224	31.2 ± 5.5, 115	26.9 ± 4.6, 88	29.4 ± 5.5, 203
Ethnicity, *n* (%)						
White	177 (81.9)	117 (78.5)	294 (80.5)	106 (76.8)	95 (73.6)	201 (75.3)
Other^2^	39 (18.1)	32 (21.5)	71 (19.5)	32 (23.2)	34 (26.4)	66 (24.7)
Annual Household Income, *n* (%)						
Did not disclose or <$50,000	40 (18.5)	26 (17.5)	66 (18.1)	23 (16.7)	24 (18.6)	47 (17.6)
50,000 to $99,999	73 (33.8)	47 (31.5)	120 (32.9)	43 (31.2)	35 (27.1)	78 (29.2)
100,000 or more	103 (47.7)	76 (51)	179 (49.1)	72 (52.2)	70 (54.2)	142 (53.2)
Education, *n* (%)						
No postsecondary degree	21 (9.7)	36 (24.2)	57 (15.6)	–	–	–
University or college graduate	107 (49.5)	74 (49.7)	181 (49.6)	–	–	–
Postgraduate training or degree	88 (40.7)	39 (26.2)	127 (34.8)	–	–	–
Energy Intake (kcal), mean ± SD	2007.3 ± 712.6	2508.6 ± 964.1	2211.9 ± 859.5	1369.4 ± 373.0	1451.2 ± 386.8	1408.9 ± 381.2

The total sample of participants from the GFHS included in this study was *n* = 365 parents and *n* = 267 children from 242 families. Missing BMI or BMI z-score data for 36 participants (11 parents and 25 children), waist circumference data for 38 participants (8 parents and 30 children), body weight data for 16 participants (*n* = 11 parents and n = 4 children) and fat mass (%) data for 205 participants (*n* = 141 parents and *n* = 64 children).

^1^BMI z-score, calculated per World Health Organization Child Growth Standards, adjusted for age and sex.

^2^Black, Chinese, Japanese, Korean, Latin American, Mixed ethnicity, South Asian, Southeast Asian, and West Asian or did not disclose.

### Distribution of energy intakes according to the degree of food processing

The dietary contribution of ultra-processed foods to total energy intake was the highest among the processed food categories, for both parents and children (Table 2). Ultraprocessed foods represented 44.3 and 41.3% of total energy intake among parents and children, respectively. Collectively, ready-to-eat meals, breads, and sweet desserts and baked goods accounted for almost half (20.6% of 44.3%) of the energy from ultra-processed foods in the parents’ diets. Breads (6.7%), sweet snacks (5.6%), and sweetened milk-based products (5.3%) were the greatest contributors of energy from ultra-processed foods in the children’s diets.

**TABLE 2:** Distribution of energy intake among NOVA food classification categories among participants in the Guelph Family Health Study (*n* = 365 parents and *n* = 267 children from 242 families).

NOVA food classification category	Parent’s energy intake		Children’s energy intake	
	Mean ± SD (kcal/day)	% Total kcal	Mean ± SD (kcal/day)	% Total kcal
**Unprocessed or minimally processed foods^1^**	**643.3 ± 404.6**	**29.1**	**497.9 ± 268.3**	**35.3**
Meat and poultry	86.0 ± 166.3	3.9	33.8 ± 85.7	2.4
Grains and flours	49.0 ± 106.2	2.2	33.6 ± 72.7	2.4
Fruit and freshly squeezed fruit juices	114.9 ± 121.3	5.2	133.3 ± 101.1	9.5
Milk and plain yogurt	54.4 ± 88.8	2.5	125.8 ± 134.6	8.9
Pasta	64.0 ± 175.3	2.9	45.0 ± 106.2	3.2
Vegetables	24.8 ± 42.0	1.1	12.1 ± 20.2	0.9
Eggs	19.1 ± 50.1	0.9	6.8 ± 23.0	0.5
Roots and tubers	11.0 ± 43.7	0.5	6.1 ± 17.0	0.4
Nuts and seeds	44.4 ± 115.6	2.0	9.8 ± 41.6	0.7
Fish and seafood	4.8 ± 31.9	0.2	3.6 ± 26.9	0.3
Legumes	14.4 ± 65.6	0.7	7.9 ± 26.9	0.6
Un-disaggregated home-made dishes^2^	149.6 ± 190.8	6.8	79.3 ± 134.5	5.6
Other unprocessed/minimally processed foods^3^	6.9 ± 27.2	0.3	0.9 ± 8.7	0.1
**Processed culinary ingredients^4^**	**57.3 ± 114.6**	**2.6**	**29.4 ± 49.4**	**2.1**
Plant oils	8.4 ± 67.1	0.4	1.6 ± 8.2	0.1
Sugars^5^	19.8 ± 41.1	0.9	15.8 ± 35.7	1.1
Animal fats	26.8 ± 65.5	1.2	11.7 ± 31.4	0.8
Other processed culinary ingredients^6^	2.3 ± 30.2	0.1	0.4 ± 6.3	0.03
**Processed foods^7^**	**531.9 ± 492.1**	**24.0**	**299.6 ± 225.4**	**21.3**
Cheese	103.3 ± 182.1	4.7	88.1 ± 126.3	6.3
Canned fruit, vegetables, other plant foods	4.0 ± 18.2	0.2	6.1 ± 20.7	0.4
Salted, smoked or canned meat or fish	31.3 ± 87.8	1.4	7.3 ± 38.2	0.5
Un-disaggregated home-made dishes^8^	233.7 ± 332.8	10.6	121.4 ± 182.6	8.6
Other processed foods^9^	159.5 ± 254.3	7.2	76.8 ± 115.1	5.4
**Ultra-processed foods^10^**	**979.4 ± 662.6**	**44.3**	**581.9 ± 308.1**	**41.3**
Pre-prepared/ready-to-eat and frozen dishes^11^	202.0 ± 398.8	9.1	53.5 ± 131.5	3.8
French fries and other potato products^12^	31.2 ± 102.6	1.4	16.0 ± 53.1	1.1
Breads	146.5 ± 212.7	6.6	93.8 ± 100.4	6.7
Soft drinks and sweetened fruit juices and drinks	38.0 ± 87.3	1.7	9.9 ± 35.5	0.7
Sweetened milk-based products^13^	66.9 ± 129.0	3.0	75.1 ± 103.0	5.3
Sweet snacks	89.6 ± 161.9	4.1	79.5 ± 109.9	5.6
Sweet desserts and baked goods	109.4 ± 233.4	4.9	51.8 ± 96.1	3.7
Sauces and spreads	84.5 ± 124.3	3.8	41.6 ± 75.6	3.0
Salty snacks	59.4 ± 120.4	2.7	51.7 ± 84.3	3.7
Reconstituted meat or fish products^14^	34.9 ± 90.7	1.6	29.6 ± 76.9	2.1
Sweetened breakfast cereals	37.0 ± 99.8	1.7	29.4 ± 58.5	2.1
Other ultra-processed foods^15^	80.2 ± 188.6	3.6	50.0 ± 106.9	3.5
**Total**	**2211.9 ± 859.5**	**100.0**	**1408.9 ± 381.2**	**100.0**

The total sample of participants from the GFHS included in this study was n = 365 parents and n = 267 children from 242 families.

^1^Unprocessed and minimally processed foods defined as naturally occurring, whole and fresh foods that undergo no or minimal industrial processing typically to preserve foods and improve palatability. Examples include vegetables, fruits, nuts, eggs, meat and milk.

^2^Made with no processed foods, but contain PCI (salts, sugars, and fats); homemade soup, omelet and baked potato.

^3^Coffee (non−presweetened, non-whitened, and non-flavored), tea (non−presweetened, non-whitened, and non-flavored), yeast, dried fruits (without added sugars) and vegetables.

^4^Processed culinary ingredients defined as substances that are used in preparation of foods to enhance flavor of meals. Examples include sugars, butter, oils, and salt.

^5^White and brown sugar, iced sugar, molasses, honey, maple syrup (100%).

^6^Vinegar, corn starch.

^7^Processed foods defined as foods that undergo some processing by combining minimally processed or unprocessed foods and processed culinary ingredients and often require minimal preparation. Examples include simple breads, cheese, salted nuts, and canned meat.

^8^Homemade mixed dishes that are not classifiable in any of the other categories. Made from adding PCI to PFs; home-made pizza with cheese, home-made lasagna.

^9^Salted, sweetened or oil roasted nuts or seeds, prepared tofu, and dried sweetened fruits (raisin).

^10^Ultra-processed foods defined as convenient foods that are a result of industrial formulations typically with five or more ingredients plus additives. Examples include Sugary drinks, chips, sweetened milk products, cereals, flavored yogurts and packaged desserts.

^11^Frozen dishes, burgers, pizzas, sandwiches and other pre-prepared products bought in fast-food outlets.

^12^Ready-to-eat and frozen French fries, onion rings, hash browns, mash potatoes and other potato products.

^13^Ice cream, chocolate milk, flavored yogurt, milkshakes.

^14^Sausages, deli-meats, meat spreads, mass-produced bacon, fish sticks.

^15^Canned soups, canned mixed dishes, cheese products, fish or seafood imitations, meal replacements, sweeteners, protein shake powder, egg substitutes, coffee whitener, meatless burgers and sausages, other sugared beverages, soy products (meatless patties, soymilk etc.).

Unprocessed or minimally processed foods represented 29.1% of total energy intake in parents’ diets and 35.3% in the children’s diets. Home-made dishes (6.8%) and fruit and freshly squeezed fruit juices (5.2%) in the parents’ diets, and fruit and freshly squeezed fruit juices (9.5%) and milk and plain yogurt (8.9%) in the children’s diets provided the greatest energy from unprocessed or minimally processed foods.

Processed foods provided 24.0 and 21.3% of total energy in the parents’ and children’s diets, respectively, with processed home-made dishes contributing the greatest source of energy for both parents (10.6%) and children (8.6%).

Processed culinary ingredients accounted for 2.6% of energy intake in the parents’ diets and 2.1% of energy intake in the children’s diets. Animal fats (1.2%) and sugars (1.1%) were the main contributors of processed culinary ingredients in the diets of parents and children, respectively.

### Associations between the degree of food processing and anthropometric indicators of obesity

For parents, ultra-processed foods (% kcal) were positively associated with BMI (β = 0.04, 95% CI: 0.01–0.07, *P* = 0.02), waist circumference (β = 0.11, 95% CI: 0.03–0.18, *P* = 0.008) and body weight (β = 0.13, 95% CI: 0.03–0.22, *P* = 0.01), with a borderline result seen for percent fat mass (β = 0.05, 95% CI: 0.00–0.11, *P* = 0.054) (Table 3). No significant associations were seen between ultra-processed food intake and anthropometric measures in children (Table 4). However, processed foods (%) were positively associated with BMI z-scores (β = 0.008, 95% CI: 0.001–0.015, *P* = 0.04) in children, but not BMI in parents. In contrast, unprocessed or minimally processed foods (% kcal) were negatively associated with waist circumference in parents (β = −0.09, 95% CI: 0.18–0.01, *P* = 0.03) and children (β = −0.03, 95% CI: 0.05–0.01, *P* = 0.01). Unprocessed or minimally processed foods (% kcal) were also negatively associated with body weight in parents (β = −0.12, 95% CI: 0.23–0.00, *P* = 0.04). No additional significant associations between processed foods or processed culinary ingredients and anthropometric measures were seen in parents or children.

**TABLE 3:** Association between the intakes of the NOVA food classification categories and anthropometric indicators of obesity^1,2^ among parents (*n* = 365 participants from 242 families).

NOVA category (% total energy intake)	BMI (kg/m^2^)	Waist circumference (cm)	Body weight (kg)	Fat mass (%)
	(*n* = 354)	(*n* = 357)	(*n* = 354)	(*n* = 224)
**Unprocessed or minimally processed foods^3^** β (95% CI) *P*-value	−0.03 (−0.07, 0.01) *P* = 0.09	−0.09 (−0.18, −0.01) *P* = 0.03	−0.12 (−0.23, 0.00) *P* = 0.04	−0.06 (−0.13, 0.01) *P* = 0.07
**Processed culinary ingredients^4^** β (95% CI) *P*-value	−0.08 (−0.18, 0.02) *P* = 0.13	0.04 (−0.40, 0.48) *P* = 0.87	−0.18 (−0.51, 0.15) *P* = 0.29	−0.16 (−0.40, 0.08) *P* = 0.20
**Processed foods^5^** β (95% CI) *P*-value	−0.02 (−0.05, 0.02) *P* = 0.36	−0.06 (−0.15, 0.03) *P* = 0.19	−0.05 (−0.15, 0.06) *P* = 0.39	−0.01 (−0.08, 0.06) *P* = 0.83
**Ultra-processed foods^6^** β (95% CI) *P*-value	0.04 (0.01, 0.07) *P* = 0.02	0.11 (0.03, 0.18) *P* = 0.008	0.13 (0.03, 0.22) *P* = 0.01	0.05 (0.00, 0.11) *P* = 0.054

The total sample of parents from the GFHS included in this study was *n* = 365. BMI z-score for 11 participants, waist circumference for eight participants, body weight for 11 participants and fat mass (%) data for 141 participants were missing and excluded from regression analyses.

^1^Results presented as linear regression coefficients (β) using generalized estimating equations with 95% confidence intervals (CI) and P-values.

^2^Models adjusted for age (years), sex, annual household income (<$50,000; $50,000–$99,999; $100,000–$149,999; $150,000 or more; Did not disclose), ethnicity [White; Other (including Black, Chinese, Japanese, Korean, Latin American, Mixed ethnicity, South Asian, Southeast Asian, and West Asian) or Did not disclose] and parental education for parent models (no postsecondary degree; postsecondary graduate; postgraduate training), or highest level of parental education for child models.

^3^Unprocessed and minimally processed foods defined as naturally occurring, whole and fresh foods that undergo no or minimal industrial processing typically to preserve foods and improve palatability. Examples include vegetables, fruits, nuts, eggs, meat, and milk.

^4^Processed culinary ingredients defined as substances that are used in preparation of foods to enhance flavor of meals. Examples include sugars, butter, oils, and salt.

^5^Processed foods defined as foods that undergo some processing by combining minimally processed or unprocessed foods and processed culinary ingredients and often require minimal preparation. Examples include simple breads, cheese, salted nuts, and canned meat.

^6^Ultra-processed foods defined as convenient foods that are a result of industrial formulations typically with five or more ingredients plus additives. Examples include Sugary drinks, chips, sweetened milk products, cereals, flavored yogurts, and packaged dessert.

**TABLE 4:** Association between the intakes of the NOVA food classification categories and anthropometric indicators of obesity^1,2^ among preschool-aged children (*n* = 267 participants from 242 families).

NOVA category (% total energy intake)	BMI Z-scores^3^	Waist circumference (cm)	Body weight (kg)	Fat mass (%)
	(*n* = 242)	(*n* = 237)	(*n* = 263)	(*n* = 203)
**Unprocessed or minimally processed foods^4^** β (95% CI) *P*-value	−0.004 (−0.011, 0.002) *P* = 0.17	−0.03 (−0.05, −0.01) *P* = 0.01	−0.009 (−0.021, 0.003) *P* = 0.15	−0.03 (−0.07, 0.01) *P* = 0.15
**Processed culinary ingredients^5^** β (95% CI) *P*-value	0.01 (−0.02, 0.04) *P* = 0.47	0.06 (−0.05, 0.16) *P* = 0.28	0.05 (0.00, 0.10) *P* = 0.07	−0.04 (−0.21, 0.13) *P* = 0.67
**Processed foods^6^** β (95% CI) *P*-value	0.008 (0.001, 0.015) *P* = 0.04	0.01 (−0.02, 0.04) *P* = 0.56	0.009 (−0.007, 0.025) *P* = 0.27	0.02 (−0.02, 0.07) *P* = 0.32
**Ultra-processed foods^7^** β (95% CI) *P*-value	−0.002 (−0.008, 0.004) *P* = 0.51	0.013 (−0.007, 0.033) *P* = 0.21	−0.001 (−0.01, 0.01) *P* = 0.92	0.01 (−0.03, 0.05) *P* = 0.59

The total sample of children from the GFHS included in this study was *n* = 267. BMI z-score for 25 participants, waist circumference for 30 participants, body weight for four participants and fat mass (%) data for 64 participants were missing and excluded from regression analyses.

^1^Results presented as linear regression coefficients (β) using generalized estimating equations with 95% confidence intervals (CI) and P-values.

^2^Models adjusted for age (years), sex, annual household income (<$50,000; $50,000–$99,999; $100,000–$149,999; $150,000 or more; Did not disclose), ethnicity [White; Other (including Black, Chinese, Japanese, Korean, Latin American, Mixed ethnicity, South Asian, Southeast Asian, and West Asian) or Did not disclose] and parental education for parent models (no postsecondary degree; postsecondary graduate; postgraduate training), or highest level of parental education for child models.

^3^BMI z-score, calculated per World Health Organization Child Growth Standards, adjusted for age and sex.

^4^Unprocessed and minimally processed foods defined as naturally occurring, whole and fresh foods that undergo no or minimal industrial processing typically to preserve foods and improve palatability. Examples include vegetables, fruits, nuts, eggs, meat, and milk.

^5^Processed culinary ingredients defined as substances that are used in preparation of foods to enhance flavor of meals. Examples include sugars, butter, oils, and salt.

^6^Processed foods defined as foods that undergo some processing by combining minimally processed or unprocessed foods and processed culinary ingredients and often require minimal preparation. Examples include simple breads, cheese, salted nuts, and canned meat.

^7^Ultra-processed foods defined as convenient foods that are a result of industrial formulations typically with five or more ingredients plus additives. Examples include Sugary drinks, chips, sweetened milk products, cereals, flavored yogurts, and packaged dessert.

## Discussion

This cross-sectional study examined associations between the degree of food processing and anthropometric measures of obesity among a sample of Canadian families with young children. The results of this study showed that ultra-processed foods accounted for a greater proportion of energy intake relative to the other less processed food categories among both parents (44%) and preschoolers (41%). Ultra-processed food intake was positively associated with a small but significant increase in BMI, waist circumference, and body weight, as well as a marginally significant increase in percent body fat in parents, but not children. Unprocessed or minimally processed foods were inversely associated with waist circumference in both parents and children, and body weight in parents only. Processed foods were positively associated with BMI z-scores in children, but no further associations with anthropometric measures were noted in parents or children.

The findings of this current study are consistent with existing research highlighting a associations between excess ultra-processed food consumption and weight gain, and conversely, unprocessed food consumption and weight loss, in adults (7). In a recent randomized controlled trial, Hall et al. (7) found that inpatients who consumed an ultra-processed diet for 2 weeks gained 0.9 ± 0.3 kg and had higher *ad libitum* energy intake relative to an unprocessed diet, whereas patients lost, on average 0.9 ± 0.3 kg while consuming an unprocessed diet. In support of our results, cross-sectional associations between ultra-processed food intake and obesity have been reported among adults from the USA (41), Canada (42), Australia (10), and Brazil (43). Findings from recent prospective studies also revealed that greater ultra-processed food intake was associated with increased incidence of obesity or weight gain among Brazilian (14), UK (13), Spanish (15), and French adults (16), as well as increased visceral fat deposition in overweight or obese older adults (aged 55–75 years old) with metabolic syndrome (17). Along with a greater risk of obesity, greater ultra-processed food intake has been associated with an increased risk of all-cause mortality and other diet-related non-communicable diseases including type 2 diabetes, hypertension and cancer (7, 44, 45). Conversely, a diet high in unprocessed or minimally processed foods (e.g., fruits, vegetables, nuts, seeds, whole grains, and fish) has been observed to have a protective effect on cardiometabolic health (46). Therefore, decreasing ultra-processed food consumption and increasing unprocessed foods in the diet may be effective health promotion strategies.

In contrast to adult studies, the association between the degree of food processing and obesity measures among children have been inconsistent (9, 47). Prospective studies found that greater ultra-processed food intake was associated with greater waist circumference in young children (aged 4–8 years old) (19), and increased adiposity trajectories tracing into early adulthood (from ages 7 to 24 years) (20). In contrast, cross-sectional studies in school-aged children and adolescents found no associations between ultra-processed or unprocessed food consumption and BMI (48) or additional indicators of obesity, including waist circumference and waist-to-height ratio (49), whereas another cross-sectional study identified a significant association between the consumption of unprocessed or minimally processed foods and excess weight among adolescents, but not ultra-processed foods (50). Our study found that energy intake from ultra-processed foods was not significantly associated with measures of obesity including BMI, waist circumference, body weight, or percent body fat in children. However, processed foods were significantly associated with BMI z-scores in children, but this small positive association may not be biologically relevant. In particular, un-disaggregated culinary preparations, which represent an important part of energy consumed by children, may be influencing the association between the consumption of processed foods and BMI z-scores. Significant inverse associations between unprocessed or minimally processed food intake and waist circumference were also identified in children. One possible explanation for the absence of this relationship in children may be due to their young age, as preschool-aged children are also rapidly growing. As the effect of ultra-processed foods was small, it may be difficult to disentangle this from normal growth (51).

While the underlying mechanisms driving the associations between the degree of food processing and risk of obesity are yet to be fully elucidated, there are several plausible mechanisms. According to the protein leverage hypothesis, the overconsumption of ultra-processed foods is driven by the need to minimize variations in absolute protein intake as a result of the reduction in dietary protein density of these foods (52). Thus, the resulting energy overconsumption from increased intake of low-protein ultra-processed foods may drive weight gain. Another possible explanation for the influence of ultra-processed foods on obesity risk is the tendency of these foods to displace nutrient-dense, unprocessed or minimally processed foods in the diet and thus, promote poor dietary patterns (53, 54). The high palatability, convenience, affordability and lower satiety of these foods may also facilitate over-eating and weight gain (53–56). A recent study proposed that compared to unprocessed foods, ultra-processed foods have a greater “energy intake rate” (a measure of energy density combined with eating rate that quantifies the rate at which energy from foods is consumed), which may further promote excess energy intakes (57). However, the proposed mechanisms by which ultra-processed foods may be related to anthropometric measures warrant further study. Further, along with dietary intake, other factors which were not examined in the current study, including physical activity, sedentary behavior, smoking status, alcohol consumption status, lipid profiles, genetics, and psychological factors, also contribute to the development of obesity and related diseases (49, 58–61).

The present study found that ultra-processed foods contributed over 40% of total energy intake in the diet of both parents and children. The relatively high intake of ultra-processed foods found in this study is also supported by previous reports (62–64). Data on household food acquisition of ultra-processed foods from countries including Canada, Brazil, Mexico, Taiwan, and Sweden showed marked increases in the contribution of ultra-processed foods, and consequent decreases in the contribution of unprocessed foods in the diet (65–69). Nationally representative dietary surveys also confirmed that ultra-processed foods represent half of the total energy in the diet of high-income countries including the USA (56%) (70), Canada (48%) (25), and UK (57%) (45). The findings of our study corroborate this data, as ultra-processed foods comprised a greater proportion of energy intake in the diet of Canadian households of middle to high-income parents (44%) and their preschool-aged children (41%), relative to the other less processed food categories. The high intakes of ultra-processed foods among preschool-aged children in our study is concerning since dietary patterns during early years of life may shape food preferences in adulthood, which could translate into the development of chronic diseases associated with poor diet (27). Further, the overall dietary intake of children in our study closely resembled the energy intake values of parents, highlighting the importance of assessing dietary patterns within the family unit. In support of our findings, studies have shown that parental dietary patterns and the food environment influence children’s feeding behaviors (28, 71). Thus, the family environment may facilitate the consumption of ultra-processed foods and should be taken into account when designing nutrition intervention strategies to elicit behavior changes (30).

This study contributes to our understanding of the associations between the degree of food processing and anthropometric indicators of obesity among a unique family based cohort of preschool-aged children. Our study explored cross-sectional associations in both parents and children, providing additional evidence for the importance of the family environment in shaping the early life dietary preferences of children. In addition, the use of individual-level dietary data in this study, as opposed to household surveys of food purchases, provides data that is more reflective of the current diet. Although this study employs a novel approach examining the association between the degree of food processing and obesity risk in a family based cohort, some limitations should be considered. Our sample included predominantly White participants from middle to high-income households and so, the generalizability of these findings to ethnically diverse and low-income households may be limited. Also, dietary reporting using the self-administered ASA24 may be subject to recall bias and underreporting of foods deemed less healthful by participants due to social desirability bias. However, these concerns are mitigated as the ASA24 is a validated dietary assessment tool for use in both adults and children. Further, culinary preparations were not disaggregated as participants were not required to report detailed recipes. Instead, culinary preparations were classified as unprocessed or minimally processed foods or processed foods, thereby potentially overestimating the dietary contribution of these categories and underestimating the contribution of processed culinary ingredients and ultra-processed foods. Due to the cross-sectional nature of our analyses, the potential causal mechanisms underlying the results of this study and the longitudinal effects of the associations between the degree of food processing and anthropometric measures in families require further study.

## Conclusion

Our study found relatively high intakes of ultra-processed foods in the diets of Canadian parents and their preschool-aged children. Ultra-processed foods were found to be positively associated with anthropometric indicators of obesity in parents, but not children. Unprocessed foods were inversely associated with abdominal obesity in both parents and children, and body weight in parents only. The overall findings from this study support the current recommendation by health professionals to reduce the consumption of ultra-processed foods and promote the consumption of unprocessed or minimally processed foods as an effort to prevent obesity. Additionally, further studies exploring prospective associations between the degree of food processing and obesity markers in a diverse family based cohort are warranted.

## Data availability statement

The datasets presented in this article are not readily available because due to Research Ethics Board restrictions and participant confidentiality, we do not make participant data publicly available. The GFHS welcomes external collaborators. Interested investigators can contact GFHS investigators to explore this option, which preserves participant confidentiality and meets the requirements of our Research Ethics Board, to protect human subjects. Requests to access the datasets should be directed to DM, davidma@uoguelph.ca.

## Ethics statement

The studies involving human participants were reviewed and approved by University of Guelph Research Ethics Board approved the study (REB #17-07-003). Written informed consent to participate in this study was provided by the participants’ legal guardian/next of kin.

## Author contributions

RA and DM designed the research, and analyzed and interpreted the data. RA conducted the research, wrote the first draft of the manuscript, and had primary responsibility for final content. All authors contributed to the review and revision of the manuscript, read, and approved the final manuscript.
